# Rituximab for the treatment of connective tissue disease–associated interstitial lung disease: A systematic review and meta-analysis

**DOI:** 10.3389/fphar.2022.1019915

**Published:** 2022-10-28

**Authors:** Linrui Xu, Faping Wang, Fengming Luo

**Affiliations:** ^1^ Department of Pulmonary and Critical Care Medicine, West China Hospital, Sichuan University, Chengdu, Sichuan, China; ^2^ Laboratory of Pulmonary Immunology and Inflammation, Frontiers Science Center for Disease-related Molecular Network, West China Hospital, Sichuan University, Chengdu, Sichuan, China; ^3^ Clinical Research Center for Respiratory Disease, West China Hospital, Sichuan University, Chengdu, Sichuan, China

**Keywords:** connective tissue disease-associated interstitial lung disease, rituximab, efficacy, safety, meta-analysis

## Abstract

**Background:** Interstitial lung disease (ILD) is a common pulmonary disease often associated with significant morbidity and mortality in patients with connective tissue diseases (CTD). Currently, no gold-standard therapies are available for CTD-ILD. Recently, several studies have proposed that rituximab (RTX) may be effective for the treatment of CTD-ILD.

**Objectives:** This study aimed to systematically evaluate the efficacy and safety of RTX for the treatment of CTD-ILD.

**Methods:** Studies were selected from PubMed, Embase, and Cochrane Library, up to 20 July 2022. Improvement and stable rates were extracted as the main outcomes and pooled using the weighted mean proportion with fixed or random-effects models, in case of significant heterogeneity (*I* ^*2*^ > 50%). Safety analysis was performed based on the adverse events reported in all of the studies.

**Results:** Thirteen studies (312 patients) were included in the meta-analysis. The follow-up durations ranged from 6 to 36 months. The pooled improvement rate was 35.0% (95% CI: 0.277–0.442), while the pooled stable rate was 59.2% (95% CI: 0.534–0.656). Anti-synthetase syndrome associated with ILD [ASS-ILD, 48.1% (95% CI, 0.373–0.620)] and idiopathic inflammatory myopathies associated with ILD [IIM-ILD, non-ASS, 47.4% (95% CI, 0.266–0.846)] had higher improvement rates than the other types. A total of 106 adverse events associated with RTX or progressive ILD were reported among the 318 patients, 55.7% of which were mild. Among 19 deaths, 17 were due to ILD progression, one to severe pulmonary arterial hypertension, and one to *Pneumocystis jirovecii* infection.

**Conclusion:** RTX, which exhibits a satisfactory safety profile, is an effective treatment option for CTD-ILD, even in patients who fail to respond to other therapies. Further randomized trials are needed to assess the efficacy of rituximab compared to other treatments for CTD-ILD.

**Systematic review registration:** PROSPERO, identifier (CRD42022363403).

## 1 Introduction

Connective tissue diseases (CTDs) are a group of disorders characterized by diverse symptoms and autoantibodies that circulate throughout the body and damage internal organs (Koo et al., 2019). Interstitial lung disease (ILD) is a common pulmonary disease that is associated with significant morbidity and mortality in patients with CTDs. The prevalence of CTD-ILD varies, depending on the underlying CTD (Marigliano et al., 2013). A high prevalence of ILD in systemic sclerosis (SSc), idiopathic inflammatory myopathies (IIM), rheumatoid arthritis (RA), and mixed CTD (MCTD), of up to 50%–60%, has been reported in previous studies. Furthermore, ILD has been reported in 25% of patients with primary Sjögren’s syndrome (pSS) and 2%–8% of patients with lupus (Lynch, 2009; Castelino and Varga, 2010; Solomon and Fischer, 2015; Mathai and Danoff, 2016). It is very important to identify ILD in CTD in the early stages and develop a proper treatment plan, which may improve prognosis.

The pathogenesis of CTD-ILD is complex and not fully understood; however, it is generally accepted that underlying immune system dysfunction, immune-mediated pulmonary inflammation, and subsequent fibrosis are crucial steps. Therefore, corticosteroids and immunosuppressive drugs are considered crucial for the treatment of CTD-ILD (Mathai and Danoff, 2016). Evidence from clinical trials suggests that immunosuppressant therapies, such as cyclophosphamide (CYC), mycophenolate mofetil (MMF), and tacrolimus, are associated with lung function improvement and ILD regression (Tashkin et al., 2006; Ge et al., 2015; Tashkin et al., 2016; Karampitsakos et al., 2022). In recent years, nintedanib, an anti-fibrotic tyrosine kinase inhibitor, has revolutionized the treatment of connective tissue diseases (Distler et al., 2019; Flaherty et al., 2019) and was approved by the United States Food and Drug Administration and the European Medicines Agency. In contrast, evidence for the efficacy of pirfenidone (another antifibrotic drug) in CTD-ILD is not equally compelling (Li et al., 2016; Acharya et al., 2020). For severe progressive CTD-ILD, intravenous CYC is considered the standard treatment (Hoyles et al., 2006). However, disease progression was observed in some patients, even with intensive therapy. Patients with SSc-ILD have a median survival duration of less than five years, and a similar poor prognosis has been observed in patients with IIM-ILD and MCTD-ILD (Goh et al., 2008). Alternative therapies may be required for patients with poor response to conventional treatment.

Biological treatments [TNF-α inhibitors (Wang et al., 2011), B-cell-targeted therapies (Sharp et al., 2016), T cell co-stimulatory molecule blockers (Fernández-Díaz et al., 2018), and immune checkpoint inhibitors (Akiyama et al., 2016)] may achieve beneficial outcomes in a proportion of patients with refractory CTD-ILD. Among them, rituximab (RTX) and TNF-α inhibitors are the most widely used biological treatments for patients with CTD-ILD (Karampitsakos et al., 2019). An observational cohort study reported better long-term survival in patients receiving rituximab than in those receiving a TNF-α inhibitor (Druce et al., 2017). RTX is a chimeric monoclonal antibody that targets CD20 expressed on pre-B and B lymphocytes, which depletes B cells from peripheral circulation for six to nine months (Leandro et al., 2006; Perosa et al., 2010). RTX has gained popularity for the management of a variety of systemic autoimmune diseases and is now approved for the treatment of RA (Smolen et al., 2017), antineutrophil cytoplasmic antibody-associated vasculitis (ANCA-associated vasculitis) (Yates et al., 2016), and immune thrombocytopenic purpura (Arnold et al., 2007). Several small studies have suggested that RTX may also be effective in CTD-ILD, with favorable responses in the treatment of patients refractory to conventional immunosuppression. RTX can be considered an effective “rescue therapy” for progressive CTD-ILD (Keir et al., 2012; Keir et al., 2014; Fitzgerald et al., 2015; Sharp et al., 2016). Most of the data supporting RTX for CTD-ILD comes from retrospective studies and small case series. To our knowledge, no systematic review has evaluated the outcomes of RTX in a population of patients with CTD-ILD. As no randomized clinical trials are available for RTX in CTD-ILD, such data are important to weigh the benefits of individual patient decision-making. However, RTX may contribute to the development and progression of pulmonary disease (Naqibullah et al., 2015). To understand and clarify the available evidence, we conducted a systematic review and meta-analysis to evaluate the efficacy and safety of RTX in patients with CTD-ILD.

## 2 Methods

We followed the Meta-Analysis of Observational Studies in Epidemiology guidelines during all stages of design, implementation, and reporting of this meta-analysis (Stroup et al., 2000). This study was registered at PROSPERO under registration number CRD42022363403.

### 2.1 Literature searching

An exhaustive literature search, both computer-assisted and manual, was performed. A systematic literature search of the PubMed, Embase and Cochrane Library was conducted, using the keywords “Rituximab”, “CD20 Antibody”, “Interstitial Lung Disease”, “Interstitial Pneumonia”, “systemic sclerosis”, “idiopathic inflammatory myopathies”, “rheumatoid arthritis”, “primary Sjögren’s syndrome”, “systemic lupus erythematosus”, and “connective tissue disease”. The last date of the search was 20 July 2022 (Supplementary Table S1).

### 2.2 Eligibility criteria

We included the studies if relevant information on patients’ characteristics, treatment interventions, and outcomes were available. The research was limited to articles published in English language. There was no restriction in study design. The inclusion criteria were: 1) the diagnosis of CTD met the accepted international criteria and the patients presented with ILD based on chest high-resolution computed tomography (HRCT) and/or lung biopsy (Bohan and Peter, 1975; Author anonymous, 1980; Arnett et al., 1988; Alarcón-Segovia and Cardiel, 1989; Hochberg, 1997; Mosca et al., 1999); 2) patients were treated with RTX were included; 3) outcomes assessed improvement rate and stable rate based on pulmonary function test (PFT). The response criteria were based on the guidance provided by American Thoracic Society/European Respiratory Society guidelines (Raghu et al., 2011). This lung response was classified into improving [increases of ≥ 10% in forced vital capacity (FVC) and/or ≥ 15% in diffusing capacity of carbon monoxide (DLCO)], worsening (decrease of ≥ 10% in FVC and/or ≥15% in DLCO, or death from progressive ILD) and stable (others that did not meet criteria for either worsening/improving). Studies with patients less than 10 were excluded. Reports only in abstract were also excluded. Adverse drug reactions and adverse events related to ILD progression were reported together in most original studies, so all reported adverse events associated with RTX treatment or progressive ILD were included for safety assessment.

### 2.3 Study selection and data extraction

Two reviewers carried out the searches, study selection and data extraction, independently. In Case of discrepancy, a consensus was reached by two reviewers. Two reviewers independently screened the titles and abstracts from the data sources based on eligibility criteria mentioned above. Then, the full texts of the potentially relevant articles were reviewed thoroughly to guarantee its eligibility criteria. We recorded the following information from the original literature (Koo et al., 2019): the first author; (Marigliano et al., 2013) year of publication; (Castelino and Varga, 2010) study design (prospective or retrospective); (Solomon and Fischer, 2015) baseline data of patients, including the number of patients that meet the inclusion criteria, type of CTD, gender, age; (Mathai and Danoff, 2016) dose and schedule of RTX; (Lynch, 2009) follow-up period; (Tashkin et al., 2006) lung response outcome; (Tashkin et al., 2016) adverse events.

### 2.4 Quality assessment

We used the modified Newcastle–Ottawa scale to make the quality assessment of observational studies (Lo et al., 2014). This scale included three parts mainly: Patient selection, comparability between study groups through design or analysis, and outcome assessment. Every study allocated a score (0–9) and a study was considered to be of high quality if it was with a score of five or more.

### 2.5 Statistical synthesis

Patients’ baseline characteristics and lung responses were analyzed from those studies enrolling ten or more patients only to avoid the extreme risk of selection and reporting biased data. The assessment criteria were the improvement and stable rate of CTD-ILD, expressed as their mean rates, together with their 95% confidence interval (95% CI). We performed subgroup analysis based on different types of CTD. The Chi-square test (*Q* statistic) and *I* ^*2*^ statistic were performed to assess heterogeneity (Higgins et al., 2003). If *p* ≥ 0.10 and/or *I* ^*2*^ ≤ 50%, the heterogeneity was recognized to be low and we would select a fixed effect model. If not, we would choose random effects. The risk of publication bias was determined by funnel plot and the Egger’s test (Egger et al., 1997). A *p-value* below 0.05 was considered statistically significant. Adverse events were extracted from all studies to provide a thorough description of safety. We used the logit transformation for meta-analyzing raw proportions with a continuity correction of 0.5 in studies with zero cell frequencies. All analyses were performed using the R programming language (package meta, version 3.6.1).

## 3 Results

### 3.1 Search results and characteristics of included studies

**FIGURE 1:**
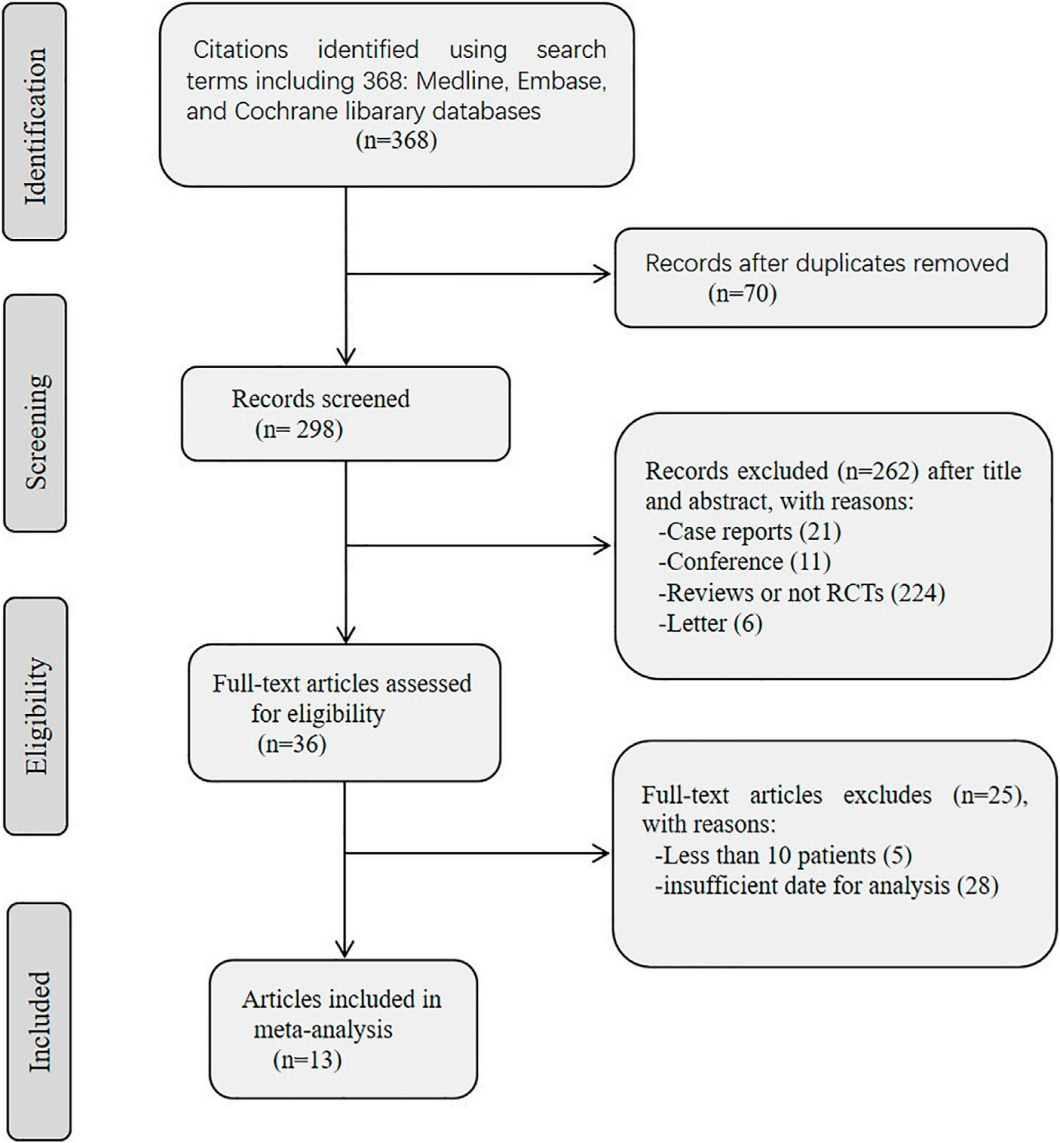
Flow diagram of study selection process for this systematic review and meta-analysis.

A flow chart of the screening process is shown in Figure 1. A total of 368 articles were identified through the database search. Of the selected studies, 70 duplicate articles were excluded using Endnote Software. After exclusion based on the titles and abstracts, 36 full-text articles were reviewed. After full-text screening, 13 publications (312 patients) were included in this systematic review. The classifications and features of the included studies are presented in Table 1. All studies were non-randomized, three (Allenbach et al., 2015; Bosello et al., 2015; Sircar et al., 2018) were prospective, and 10 (Sem et al., 2009; Keir et al., 2014; Lepri et al., 2016; Sharp et al., 2016; Md Yusof MYKabia et al., 2017; Sari et al., 2017; Doyle et al., 2018; Duarte et al., 2019; Narváez et al., 2020a; Narváez et al., 2020b) were retrospective. In total, 312 patients were diagnosed with CTD-ILD, including RA, SSc, IIM, SLE, pSS, UCTD, and MCTD. Among the 13 included studies (Sem et al., 2009; Keir et al., 2014; Allenbach et al., 2015; Bosello et al., 2015; Lepri et al., 2016; Sharp et al., 2016; Md Yusof MYKabia et al., 2017; Sari et al., 2017; Doyle et al., 2018; Sircar et al., 2018; Duarte et al., 2019; Narváez et al., 2020a; Narváez et al., 2020b), 157 patients with CTD-ILD (212/312, 67.9%) were refractory to conventional treatments. The studies were performed in the United Kindom (*n* = 4) (Keir et al., 2014; Sharp et al., 2016; Md Yusof MYKabia et al., 2017; Duarte et al., 2019), Spain (*n* = 2) (Narváez et al., 2020a; Narváez et al., 2020b), French (*n* = 1) (Allenbach et al., 2015), United States (*n* = 1) (Doyle et al., 2018), Turkey (*n* = 1) (Sari et al., 2017), Norway (*n* = 1) (Sem et al., 2009), India (*n* = 1) (Sircar et al., 2018), and Italy (*n* = 1) (Bosello et al., 2015), and there was only one multicenter study (Lepri et al., 2016). There were 208 (208/312, 66.7%) patients treated with RTX (1,000 mg on day 0 and day 14/day 15), one (1/312, 0.3%) patient treated with RTX (700 mg on day 0 and day 14) and one (1/312, 0.3%) patient treated with RTX (1,000 mg on day 0, day 15, and month 6), while the treatment plans were not available in other studies. Treatments before RTX varied among the studies. CYC, MMF, azathioprine (AZA), and steroids were the most commonly used treatments, while less common treatments included intravenous immunoglobulin (IVIg), TNFi, and tacrolimus. The follow-up period was 6–36 months (Table 1). Quality assessment using the Newcastle–Ottawa scale is shown in Table 2.

**TABLE 1:** Baseline clinical characteristics of included studies.

Study	Study design	Country	Population	Patient (*n*)	Sex (F (%)	Mean age (yrs)	Evaluation criteria	Rituximab therapy	Follow-up (months)	Quality score
Sem et al, (2009)	Retrospective study	Norway	AS-ILD	11	63	59 (23–66)	PFT、HRCT	Rituximab (1,000 mg) on D0 and D14 (*n* = 10)	6	7
								Rituximab (700 mg) on D0 and D14 (*n* = 1)		
Keir et al, (2014)	Retrospective study	United Kingdom	CTD-ILD	32	33	52.5 ± 10.9	PFT	Rituximab (1,000 mg) on D0 and D14	6–12	7
Allenbach et al, (2015)	Prospective study	French	AS-ILD	10	20	51 (18–57)	PFT、HRCT、SF-36	Rituximab (1,000 mg) on D0, D15 and M6	12	7
Bosello et al, (2015)	Prospective study	Italy	SSC-ILD	14	85	41.4 ± 13.1	PFT、HRCT	Rituximab (1,000 mg) on D0 and D14	12	8
Lepri et al, (2016)	Retrospective study	NA	CTD-ILD	42	75	NA	PFT	NA	12	7
Sharp et al, (2016)	Retrospective study	United Kingdom	CTD-ILD	24	66	51.4 ± 14.9	PFT、HRCT	Rituximab (1,000 mg) on D0 and D14	6–12	7
Yuzaiful (2017)	Retrospective study	United Kingdom	RA-ILD	43	64	64 (59–72)	PFT、HRCT	Rituximab (1,000 mg) on D0 and D14	6–12	7
Sari et al, (2017)	Retrospective study	Turkey	SSC-ILD	14	92	53.2 (46.8–55.5)	PFT	NA	6-?	7
Doyle et al, (2018)	Retrospective study	United States	AS-ILD	22	80	49 ± 12	PFT、HRCT	NA	12–36	8
Sircar et al, (2018)	Prospective study	India	SSC-ILD	30	83	34.67 ± 8.13	PFT、HRCT	Rituximab (1,000 mg) on D0 and D15	6	8
Duarte et al, (2019)	Retrospective study	United Kingdom	RA-ILD	15	66	NA	PFT、HRCT	NA	6–36	7
Javier (2020)	Retrospective study	Spain	SSC-ILD	24	87.5	58.0 ± 14.0	PFT、HRCT	Rituximab (1,000 mg) on D0 and D15	6–24	7
Javier (2020)	Retrospective study	Spain	RA-ILD	31	58	61.0 ± 12.0	PFT、HRCT	Rituximab (1,000 mg) on D0 and D15	6–24	7

Abbreviations: RA-ILD, Rheumatoid Arthritis-Associated Interstitial Lung Disease; AS-ILD: Anti-synthetase Syndrome-Associated Interstitial Lung Disease. CTD-ILD: Connective Tissue Disease-Associated Interstitial Lung Disease; SSC-ILD: Systemic Sclerosis-Associated Interstitial Lung Disease. PFT: pulmonary function test; HRCT: High-Resolution Computed Tomography; SF-36: 36-Item Short-Form Health Survey. N/A: not available.

**TABLE 2:** Quality assessment of included studies by Newcastle–Ottawa scale (score).

Study	Selection				Comparability	Outcome			
	Representativeness of the exposed cohort	Selection of the non-exposed cohort	Ascertainment of exposure	Outcome present at start of study		Assessment of outcome	Adequate follow-up	Complete follow-up	Quality score
Sem et al, (2009)	★	NO	★	★	★	★	★	★	★★★★★★★
Keir et al, (2014)	★	NO	★	★	★	★	★	★	★★★★★★★
Allenbach et al, (2015)	★	NO	★	★	★	★	★	★	★★★★★★★
Bosello et al, (2015)	★	★	★	★	★	★	★	★	★★★★★★★★
Lepri et al, (2016)	★	NO	★	★	★	★	★	★	★★★★★★★
Sharp et al, (2016)	★	NO	★	★	★	★	★	★	★★★★★★★
Yuzaiful (2017)	★	NO	★	★	★	★	★	★	★★★★★★★
Sari et al, (2017)	★	NO	★	★	★	★	★	★	★★★★★★★
Doyle et al, (2018)	★	★	★	★	★	★	★	★	★★★★★★★★
Sircar et al, (2018)	★	★	★	★	★	★	★	★	★★★★★★★★
Duarte et al, (2019)	★	NO	★	★	★	★	★	★	★★★★★★★
Javier (2020)	★	NO	★	★	★	★	★	★	★★★★★★★
Javier (2020)	★	NO	★	★	★	★	★	★	★★★★★★★

### 3.2 Response rate

#### 3.2.1 Improvement rate

**FIGURE 2:**
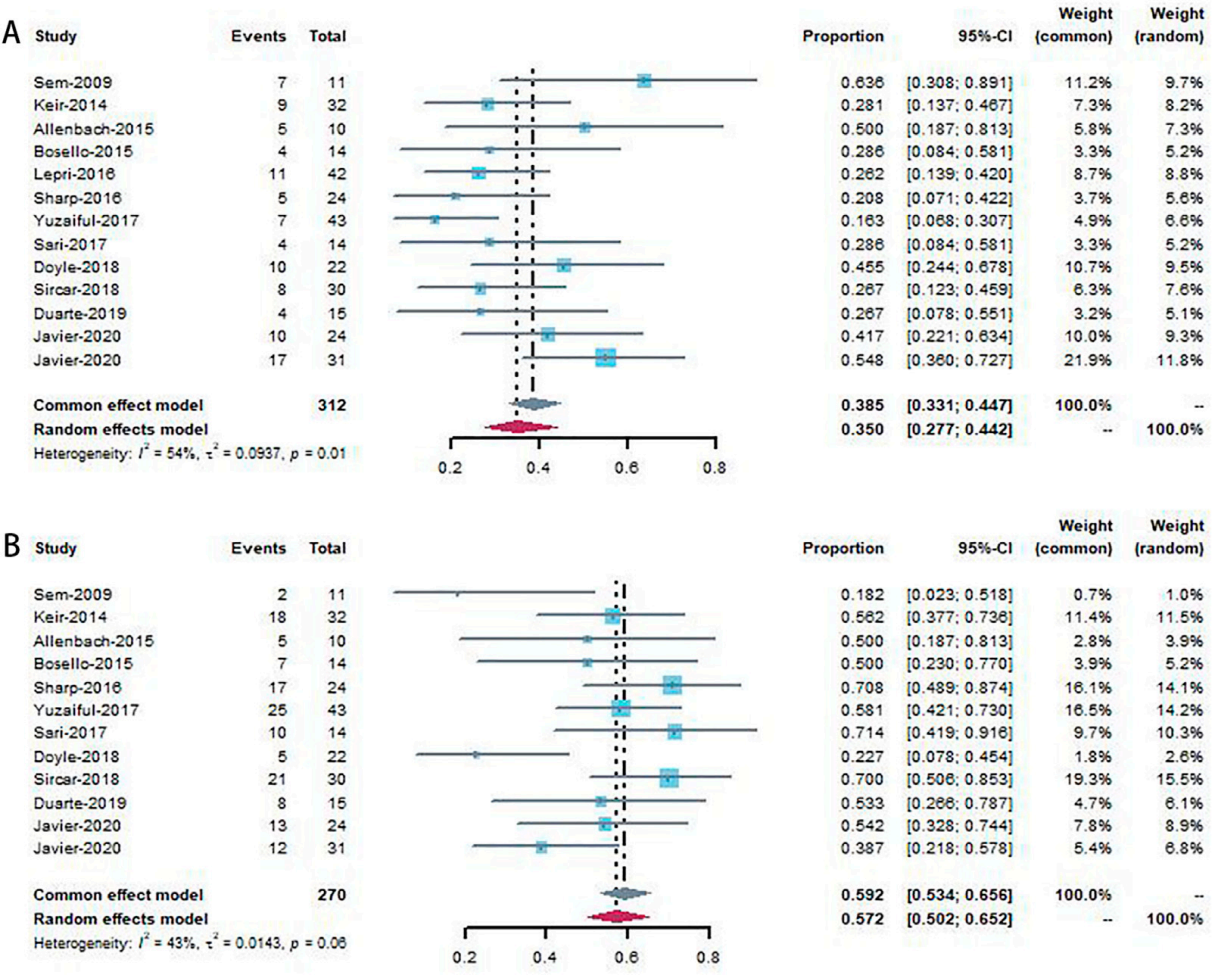
Forest plot showing improvement rate **(A)** and stable rate **(B)** to patients using rituximab.

The pooled improvement rate was evaluated in 13 studies that included 312 patients with CTD-ILD. A total of 101 patients (101/312, 32.4%) reported improved lung function after RTX in all studies. The improvement rate ranged from 16% to 64%, and the pooled rate was 35.0% (95% CI, 0.277–0.442), with high heterogeneity (*I* ^*2*^ = 54%, *p* = 0.01) (Figure 2A).

**FIGURE 3:**
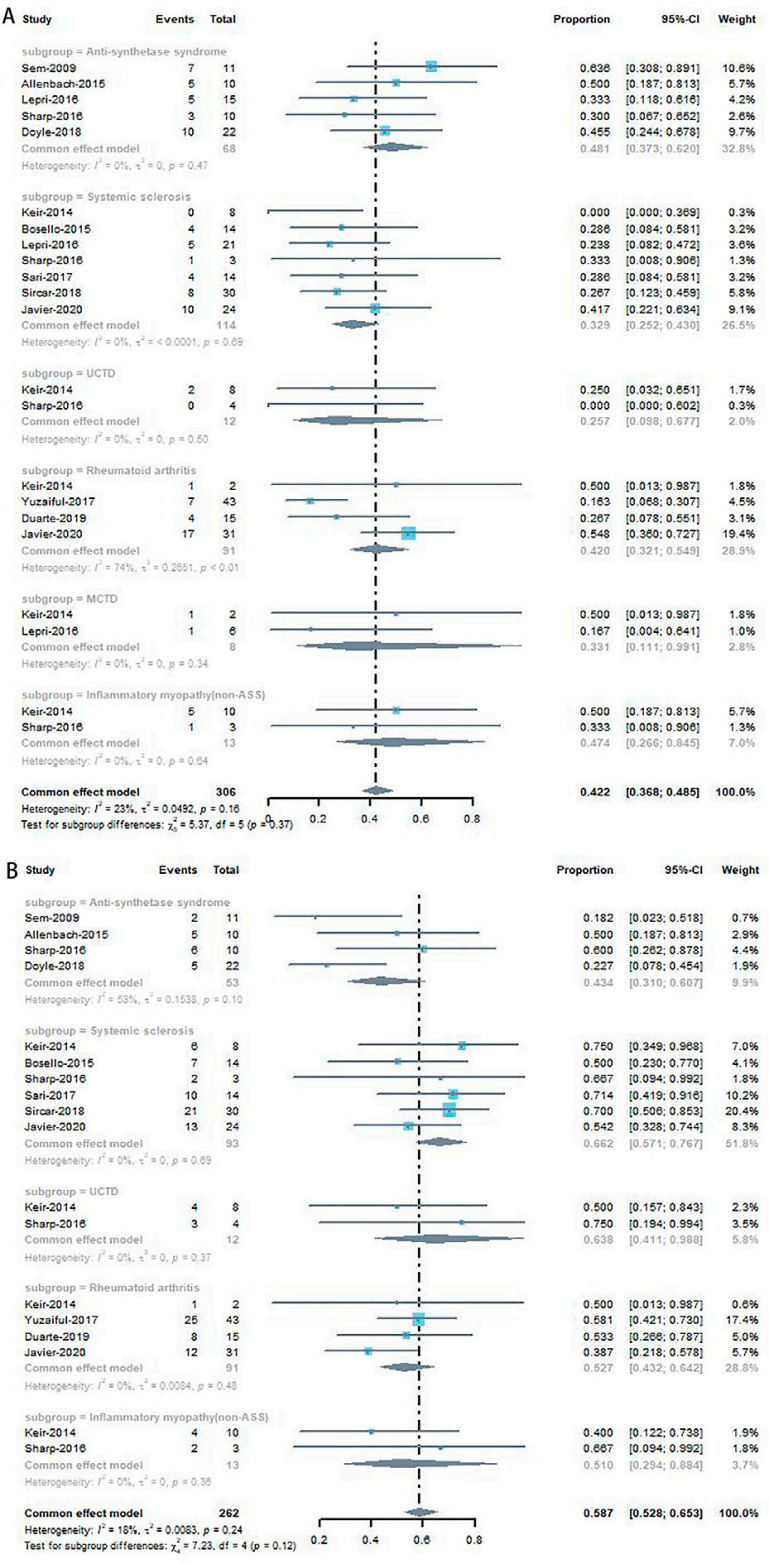
Subgroup analyses of improvement rate **(A)** and stable rate **(B)** in CTD-ILD studies.

Forest plots for the subgroup analyses of studies involving anti-synthetase syndrome (ASS)-ILD, IIM (non-ASS)-ILD, MCTD-ILD, RA-ILD, SSc-ILD, and UCTD-ILD are shown in Figure 3A. ASS-ILD, IIM (non-ASS)-ILD, MCTD-ILD, SSc-ILD and UCTD-ILD were associated with improvement rates of 48.1% (95% CI, 0.373–0.620), 47.4% (95% CI, 0.266–0.845), 33.1% (95% CI, 0.111–0.991), 32.9% (95% CI, 0.252–0.430) and 25.7% (95% CI, 0.098–0.677) respectively, without heterogeneity, except for RA (17% (95% CI, 0.04–0.48), *I* ^*2*^ = 74%, *p*＜0.01).

#### 3.2.2 Stable rate

Twelve studies were included in the analysis of lung function stability (Figure 2B). The stability rates ranged from 18% to 71%, and the pooled rate was 59.2% (95% CI, 0.534–0.656) with low heterogeneity (*I* ^*2*^ = 43%, *p* = 0.06).

The forest plot for the subgroup analyses is shown in Figure 3B. IIM (non-ASS)-ILD, RA-ILD, SSc-ILD, and UCTD-ILD were associated with stable rates of 51.0% (95% CI, 0.294–0.884), 52.7% (95% CI, 0.432–0.642), 66.2% (95% CI, 0.571–0.767), and 63.8% (95% CI, 0.411–0.988), respectively, without heterogeneity, except for ASS-ILD [43.4% (95% CI, 0.310–0.607), *I* ^*2*^ = 53%, *p* = 0.10].

### 3.3 Adverse events

All patients treated with RTX were evaluated, some of whom were not included in the pooled analysis of lung response outcomes because lung function data and survival conditions were unavailable. Twelve (Sem et al., 2009; Keir et al., 2014; Allenbach et al., 2015; Bosello et al., 2015; Lepri et al., 2016; Sharp et al., 2016; Sari et al., 2017; Doyle et al., 2018; Sircar et al., 2018; Narváez et al., 2020a; Narváez et al., 2020b) studies reported adverse events. Among them, one study (Keir et al., 2014) reported three deaths due to respiratory failure secondary to ILD progression, but did not report other adverse events. One study (Duarte et al., 2019) did not provide any relevant information. We evaluated adverse events related to RTX treatment or progressive ILD according to the Common Terminology Criteria for Adverse Events (CTCAE), which are shown in Table 3. A total of 318 patients from 12 studies were included. A total of 55.7% of the adverse events were mild-to-moderate (grade 1–2), including a mild infection that was treated with oral antibiotics without hospitalization (*n* = 44), fever (*n* = 6), infusion reactions (*n* = 5), fatigue (*n* = 3), and cardiac involvement with arrhythmia (*n* = 1). Among grade 3–4 events, 28 adverse events occurred, including infection requiring hospitalization (*n* = 23), serum sickness (*n* = 2), gastrointestinal complications requiring surgery (*n* = 2), and anaphylaxis (*n* = 1). Nineteen deaths were reported in 318 patients: 17 due to respiratory failure secondary to ILD progression, one with severe pulmonary arterial hypertension, and one with *Pneumocystis jirovecii* infection.

**TABLE 3:** Adverse events observed after rituximab infusion in CTD-ILD patients.

Study	CTD-ILD patients, *n*	Adverse events, *n*	Adverse events, (*n*)		
			Grade1–2	Grade3-4	Grade5
Sem et al, (2009)	11	8	7	0	1
Keir et al, (2014)	33	3	NA	NA	3
Allenbach et al, (2015)	12	6	6	0	0
Bosello et al, (2015)	14	6	6	0	0
Lepri et al, (2016)	44	12	10	2	0
Sharp et al, (2016)	24	1	0	0	1
Yuzaiful (2017)	56	24	0	15	9
Sari et al, (2017)	14	1	0	1	0
Doyle et al, (2018)	25	13	9	3	1
Sircar et al, (2018)	30	13	11	1	1
Duarte et al, (2019)	26	NA	NA	NA	NA
Javier (2020)	24	9	5	3	1
Javier (2020)	31	10	5	3	2

Abbreviations: CTD-ILD: Connective Tissue Disease-Associated Interstitial Lung Disease; NA: not available. Grade1-2: Mild to moderate. Grade 3: Severe but not immediately life-threatening. Grade 4: Life-threatening consequence. Grade 5: Death.

### 3.4 Sensitivity analysis

**FIGURE 4:**
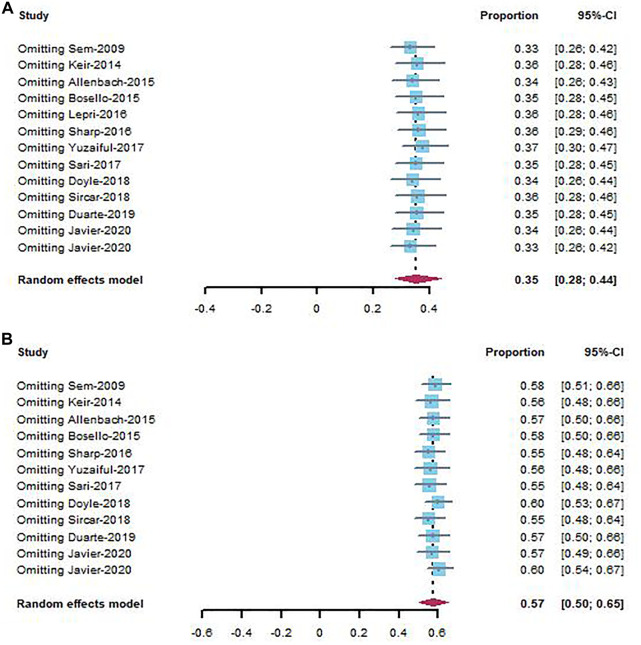
Sensitivity analysis of improvement rate **(A)** and stable rate **(B)** in CTD-ILD studies.

Sensitivity analysis was performed by removing individual studies one by one from the pooled results with high heterogeneity. The pooled analysis of improvement rate and stable rate did not change significantly when studies were omitted, indicating that our combined results are reliable (Figure 4).

### 3.5 Publication bias

**FIGURE 5:**
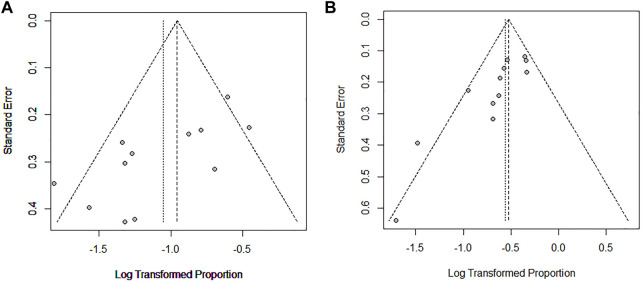
Egger funnel plot of improvement rate **(A)** and stable rate **(B)** in CTD-ILD studies.

We used the Egger’s test and funnel plots to evaluate the publication bias in studies included. The results of the Egger’s test showed no evidence of publication bias in the studies on improvement rate (*p* = 0.17) (Figure 5A) and stable rate (*p* = 0.21) (Figure 5B). This was consistent with the shape of funnel plots which had a good symmetry.

## 4 Discussion

To the best of our knowledge, this is the first meta-analysis of observational studies on the efficacy and safety of RTX in CTD-ILD. Considering that most of the involved patients were refractory to conventional treatments and had progressive ILD, improving or stabilizing lung function was regarded as an effective response. In our results, the improvement rate was estimated to be 35.0% in 312 patients with CTD-ILD, while the stable rate was 59.2%. This result indicates that RTX was an effective treatment option for patients with CTD-ILD based on the lung function test, even in those who failed to respond to other conventional therapies, such as CYC. A total of 106 adverse events associated with rituximab treatment or progressive ILD were reported among the 318 patients. Most were mild, indicating that RTX was tolerated in most patients.

An optimal management for CTD-ILD has not been established because of the relative rarity of the disease and the high variability in disease presentation. Currently, corticosteroids are widely used to manage CTD-ILD (Kowal-Bielecka et al., 2009; Maher, 2014). Other immunosuppressants, including alkylating agent CYC, purine analog azathioprine, antifolate drug methotrexate, inosine monophosphate dehydrogenase inhibitor mycophenolate mofetil, and calcineurin inhibitors ciclosporin and tacrolimus, are also used in the management of CTD-ILD. Among these, CYC is the most well studied for CTD-ILD. Two multicenter randomized studies reported that CYC treatment is associated with an improved FVC trend (Hoyles et al., 2006; Tashkin et al., 2006). A recent open-label, randomized, controlled trial for SSc-ILD that compared the efficacy of RTX and CYC found that RTX improved FVC% while CYC did not after six months of treatment (Sircar et al., 2018). Stone et al. found that rituximab therapy was inferior to daily cyclophosphamide treatment in inducing remission of relapsing disease in severe ANCA-associated vasculitis (Stone et al., 2010). The efficacy and safety reported in these trials suggest that RTX may be considered a first-line therapy (Roofeh et al., 2019). In our meta-analysis, most patients were treated with other immunosuppressants before RTX treatment; however, the efficacy was limited. Stability or improvement of PFT was seen in the majority of patients after RTX therapy in our study, which indicates RTX as a choice in the management of refractory CTD-ILD. However, due to the absence of controlled studies, the present meta-analysis was unable to draw firm conclusions about the difference in efficacy between RTX therapies and other drugs.

Nowadays the treatment of CTD-ILD has a more extended measure with the presence of antifibrotic treatments. The main antifibrotic drugs, pirfenidone (King et al., 2014) and nintedanib (Richeldi et al., 2011), have been proved efficacious for the management of IPF by phase II and III clinical trials. Given the fact that CTD-ILD share many imaging and histopathological characters with IPF (61), of particular interest for rheumatologists are the effectiveness of antifibrotic treatments in CTD-ILD. Nintedanib proved efficacious in reducing the annual rate of decline in FVC in patients with SSc-ILD (12) in the phase III SENSCIS trial, and in treating patients who have fibrosing ILDs with a progressive phenotype (including CTD-ILDs) in the phase III INBUILD study (Flaherty et al., 2019). Pirfenidone showed a modest effectiveness in the decline of FVC in progressive fibrotic CTD-ILD based on a small sample size (Guenther et al., 2019). However, no clinical trials are performed to compare the efficacy between RTX therapies and antifibrotic treatments. Patients involved in our meta-analysis are not received antifibrotic treatment. Further studies are needed to explore the efficacy between RTX therapies and antifibrotic treatments in patients with CTD-ILD who failed to respond to other conventional therapy.

No consensus criteria for assessing the treatment efficacy of CTD-ILD are currently available. Some studies were excluded from our pool analysis because of different assessment methods that focused on the efficacy of RTX. These excluded studies assessed efficacy by comparing mean/median FVC and/or DLCO pre- and post-RTX treatment using the Student *t*-test or Wilcoxon’s rank sum test. Daoussis et al. (2012) found a significant increase of FVC and DLCO at two years after RTX treatment, compared to baseline (FVC 77.13 ± 7.13 vs. 68.13 ± 6.96; DLCO 63.13 ± 7.65 vs. 52.25 ± 7.32) for SSc-ILD (Daoussis et al., 2012). Two studies focusing on ASS revealed that FVC increased from 66% to 74% and 58%–72%, while DLCO increased from 39% to 59% and 41%–48% after RTX therapy, respectively (Marie et al., 2012; Andersson et al., 2015). A study by Fui found that FVC and DLCO percentages stabilized after RTX treatment (Fui et al., 2019). Our analysis supported these positive findings. In addition, Doyle et al. (2018) found that corticosteroids were stable or decreased in 88% of patients with CTD-ILD one year after RTX treatment, with an average drop of 6 mg, which could be helpful for decreasing the side effects of corticosteroids.

There was medium heterogeneity in the improvement rate (*I* ^*2*^ = 54%) and stability rate (*I* ^*2*^ = 43%). Subgroup analysis revealed that IIM-ILD (non-ASS) (improvement rate, 47.4%) and ASS-ILD (improvement rate, 48.1%) had higher improvement rates than the others. This adds to the weight of the evidence regarding the heterogeneity of CTD-ILD. However, the pathogenesis of CTD-ILD is not fully understood, and the mechanism by which IIM-CTD responds better to RTX therapy than the other types of CTD-ILD is uncertain. Further research that investigates on the pathogenic mechanisms of CTD-ILD is required.

RTX is a drug that leads to the depletion of B-cells, the mechanism of which remains unknown, but some evidence supports the possibility that B cell function may contribute to the pathogenesis of CTD-ILD. B cell infiltration was found in lung biopsies of 11 patients with SSc-associated ILD (Lafyatis et al., 2007). A study that focused on bronchoalveolar lavage (BAL) fluid found that ILD progression was associated with a higher B-cell percentage in BAL fluid in 73 patients with SSc-ILD (De Santis et al., 2012). It is well known that B cells are a source of autoantibodies, some of which may contribute to the pathogenesis of CTD (Baroni et al., 2006). In addition, RTX can indirectly affect other immune cells, such as T cells, to ““normalizeˮ auto-reactive T cells (Sfikakis et al., 2005; Sfikakis et al., 2007). The repopulation of the B-cell line following RTX tends to be antigenically inexperienced, which suggests that the degree of immune system resetting may contribute to the therapeutic effect (Anolik et al., 2007).

A study conducted by Gagiannis et al. (2020) found overlapping serological, clinical, radiologic, and histopathological features of severe COVID-19 and lung manifestations of autoimmune disease (CTD-ILD). Another study (Narváez et al., 2020a) included patients between January 2010 and December 2019; some of whom may have had COVID disease, as the initial COVID-19 outbreak was reported in 2019. In addition, rituximab use was significantly associated with a higher risk of COVID-19 in CTD-ILD (de Oliveira et al., 2022) included patients between January 2010 and December 2019; some of whom may have had COVID disease, as the initial COVID-19 outbreak was reported in 2019. In addition, rituximab use was significantly associated with a higher risk of COVID-19 in CTD-ILD.

RTX is well-tolerated and safe for CTD-ILD. The adverse events were mainly infectious, most of which were mild and resolved soon after antibiotic treatment. Although there were 19 deaths reported in our analysis, most were due to progressive ILD. Among them, nine deaths with a median DLCO of 41% predicted pre-RTX were reported [36], which indicates severely impaired lung function. However, the safety of RTX warrants further investigation.

This meta-analysis has several limitations. First, the number of patients included was small, and all studies were observational. The small sample size may have influenced the strength of our study. Second, the sex ratio discrepancy among the studies varied due to the small number of included patients, and the female predominant phenomenon may affect the result of the treatment effect analysis in males. Third, all studies failed to compare the efficacy of RTX with other drugs, so we could not provide an unbiased head-to-head comparison of the treatment effects. Fourth, HRCT was not analyzed as an evaluation index, which can be considered another assessment of the efficacy of RTX. It is difficult to pool these data because the criteria for the assessment of HRCT varied among the eligible studies.

## 5 Conclusion

In this systematic review and meta-analysis, RTX was found to be an effective treatment option for CTD-ILD according to the assessment of improvement and stability rates based on PFT, even in those who failed to respond to other conventional therapies. Our study revealed that patients with IIM-CTD (non-ASS) or ASS-ILD responded better to rituximab than those with other CTD-ILDs. Regarding side effects, most patients showed good tolerance to RTX. Considering these limitations, prospective randomized trials are needed to assess the efficacy of rituximab compared to other drugs in CTD-ILD. Consensual criteria based on PFT and HRCT for the assessment of CTD-ILD treatment efficacy should be established in the future.

## Data Availability

The original contributions presented in the study are included in the article/Supplementary Material, further inquiries can be directed to the corresponding authors.
