# Supplementary material for: Rituximab for the treatment of connective tissue disease–associated interstitial lung disease: A systematic review and meta-analysis
Source: Front Pharmacol. 2022 Oct 28;13:1019915. doi: 10.3389/fphar.2022.1019915 (PMC9650441; doi:10.3389/fphar.2022.1019915)
Supplement: Supplementary file 1 [file Table1.docx]

Supplementary Material

# Supplementary Table

**Table S1.** Search terms of pubmed

| **#1** | Search (("Rituximab"[Mesh]) OR (((((((((CD20 Antibody, Rituximab[Title/Abstract]) OR Rituximab CD20 Antibody[Title/Abstract]) OR Mabthera[Title/Abstract]) OR IDEC-C2B8 Antibody[Title/Abstract]) OR IDEC C2B8 Antibody[Title/Abstract]) OR IDEC-C2B8[Title/Abstract]) OR IDEC C2B8[Title/Abstract]) OR GP2013[Title/Abstract]) OR Rituxan[Title/Abstract])) |
| --- | --- |
| **#2** | Search (((((((((((("Lung Diseases, Interstitial"[Mesh]) OR Interstitial Lung Disease[Title/Abstract]) OR Lung Disease, Interstitial[Title/Abstract]) OR Lung Disease, Interstitial[Title/Abstract]) OR Diffuse Parenchymal Lung Diseases[Title/Abstract]) OR Pneumonia, Interstitial[Title/Abstract]) OR Interstitial Pneumonia[Title/Abstract]) OR Interstitial Pneumonias[Title/Abstract]) OR Pneumonias, Interstitial[Title/Abstract]) OR Pneumonitis, Interstitial[Title/Abstract]) OR Interstitial Pneumonitides[Title/Abstract]) OR Interstitial Pneumonitis[Title/Abstract]) OR Pneumonitides, Interstitial[Title/Abstract] |
| **#3** | Search (((((((((("Connective Tissue Diseases"[Mesh]) OR Connective Tissue Disease[Title/Abstract]) OR Disease, Connective Tissue[Title/Abstract]) OR Diseases, Connective Tissue[Title/Abstract]) OR Rheumatic Diseases[Title/Abstract]) OR systemic sclerosis[Title/Abstract]) OR rheumatoid arthritis[Title/Abstract]) OR idiopathic inflammatory myopathies[Title/Abstract]) OR primary Sjogren's syndrome[Title/Abstract]) OR anti-synthetase syndrome[Title/Abstract]) OR systemic lupus erythematosus[Title/Abstract] |
| Final search term: #1 AND #2 AND #3 | |
